# The panorama of future sick-leave diagnoses among young adults initially long-term sickness absent due to neck, shoulder, or back diagnoses. An 11-year prospective cohort study

**DOI:** 10.1186/1471-2474-10-84

**Published:** 2009-07-14

**Authors:** Marjan Vaez, Jan Hagberg, Kristina Alexanderson

**Affiliations:** 1 Department of Clinical Neuroscience, Division of Personal Injury Prevention, Karolinska Institutet, Stockholm, Sweden; 2 Department of Health and Society, Division of Social Medicine and Public Health, Linköping University, Linköping, Sweden

## Abstract

**Background:**

Little is known about future sick-leave diagnoses among individuals on long-term sickness absence. The aim of this study was to describe the panorama of sick-leave diagnoses over time among young adults initially sick-listed for ≥ 28 days due to back, neck, or shoulder diagnoses

**Methods:**

An 11-year prospective population-based cohort study including all 213 individuals in a Swedish municipality who, in 1985, were aged 25–34 years and had a new sick-leave spell ≥ 28 days due to neck, shoulder, or back diagnoses.

**Results:**

Over the 11-year period, the young adults in this cohort had 176,825 sick-leave days in 7,878 sick-leave periods (in 4,610 sick-leave spells) due to disorders in 17 of the 18 ICD-8 diagnostic categories (International Classification of Diseases, Revision 8). Musculoskeletal or mental diagnoses accounted for most of the sick-leave days, whereas most of the sick-leave periods were due to musculoskeletal, respiratory, or infectious disorders, or to unclassified symptoms. Most cohort members had had four to eight different sick-leave diagnoses over the 11 years, although some had had up to 11 diagnoses. Only two individuals (1%) had been sickness absent solely due to musculoskeletal diagnoses.

**Conclusion:**

Although the young adults initially were sick listed with back, neck, or shoulder diagnoses, their sickness absence during the follow up were due to a wide variety of other medical diagnoses. It might be that the ill-health content of sickness absence due to back pain is greater than usually assumed. More research on prognoses of sick-leave diagnoses among long-term sick listed is warranted.

## Background

In Sweden, as well as in many other industrialized countries, sickness absence is considered to be a major public health issue [1-4]. Until recently, musculoskeletal disorders have been the most common reason for sickness absence and disability pension (DP) in the working population [1]. Even though these medical conditions are likely to be recurrent and long-lasting, and can often result in DP [5], very little is known about the prognoses of the patients sick listed due to musculoskeletal disorders [1]. In fact, there is even a lack of applicable methods to describe such prognoses due to several methodological challenges. One major difficulty in this context is associated with the recurrence of sickness absence and the uneven distribution of incidence and duration of sick leave in general [6]. The variation of sick-leave diagnoses between, as well as within sick-leave periods is another important issue that needs to be considered. Patients with this type of disorders (e.g., low-back, neck, or shoulder pain) very often seek clinical help and issuing a sick note is a very common measure taken by physicians. Arriving at prognoses of work capacity for those individuals is a daily task for many of the general practitioners and orthopaedic surgeons whose job it also is to, with their patient, discuss the pros and cons of being sickness absent [1-5,7-9]. Furthermore, determining the work ability of such patients is an even greater challenge for the physicians concerned [1].

According to a systematic review of the studies on sickness absence [1], despite the magnitude of the problem, few studies have focused on this aspect, and the majority of those that have been performed are of poor scientific quality. The review also indicated that research in this area is still underdeveloped with regard to both theories and methods, and, in particular, there is a need for prospective population-based studies, as well as investigations that consider the medical aspects of sickness absence. Such information is needed by physicians, sickness insurance staff, and employers in order to allow them to implement optimal measures to help their patients/clients/employees who are sick listed due to specific diagnoses.

Despite the importance of the medical conditions of individuals on sick leave, very few epidemiological studies have prospectively followed patients over time with respect to the diagnoses given for their sickness absences. We found only one report published in Norwegian [10] that described changes in sick-leave diagnoses. A major reason for few such studies is a general lack of data on diagnosis-specific sick leave over time. In the present study, we used unique data comprising such information on a population-based cohort of young adults who initially were sick-listed ≥ 28 days due to neck, shoulder, or back diagnoses. In our earlier investigations based on this cohort [11-14], we found that the study population was at high risk of DP. This is illustrated by the finding that, over a period of 11 years, such benefits were granted to 22% of these young adults and a large proportion of those individuals (76%) was granted DP due to musculoskeletal diagnoses, while 15% were granted DP due to mental diagnoses [15]. As early as during the first year of the study period, there was a considerable difference in the level of sickness absence between those who later were and those who were not granted DP [12]. For the latter group, the number of sick-leave days was substantially lower during follow-up.

Our previous findings suggested that the number and pattern of changes in sick-leave diagnoses differed between those who were and those who were not granted DP [13]. More specifically, those granted DP, had had fewer changes in sick-leave diagnoses. More knowledge is needed regarding the patterns of sick-leave diagnoses, as well as the approaches used to describe and measure such patterns. The current study was conducted to further pursue the analyses.

Thus, in order to further pursue analyses of prognoses of sick-leave among long-term sickness absent individuals, *the aim *of this study was to describe the panorama of sick-leave diagnoses over time among young adults initially sick-listed for ≥ 28 days due to neck, shoulder, or back diagnoses. Three specific study questions were addressed:

1. Which sick-leave diagnoses were most frequent when different measures of sickness absence were employed?

2. How common was it to have only one, few, or several sick-leave diagnoses over the 11 years?

3. Did individuals with several different sick-leave diagnoses also have mental sick-leave diagnoses?

## Methods

A prospective cohort study was conducted including all the individuals who, in 1985, fulfilled the following criteria: aged 25–34; registered as living in the Municipality of Linköping, Sweden (population. 132,000), and had a new sick-leave spell ≥ 28 days due to a neck, shoulder, or back diagnoses. The third criterion included the following diagnoses: displacement of intervertebral lumbar disc, tendinitis, lumbago, other deformities, spondylosis, sciatica, periarthritis humeroscapularis, myalgia, cervicalgia, and cervicobrachialgia. Individuals with diagnoses of arthroses, rheumatic, or inflammatory musculoskeletal diseases, or pregnancy-related conditions were excluded.

The Swedish Social Insurance Offices were not permitted to store data on sick-leave diagnoses. Therefore, to be able to identify the subjects for our study, we used a large research database covering all new physician-certified sick-leave spells > 7 days in 1985 in this municipality [14,16,17]. The inclusion criteria for the individuals identified in that manner were subsequently checked manually by examining their sickness certificates at the relevant local Social Insurance Offices, which indicated that 213 individuals (61% women) satisfied the specifications. There were no gender differences in mean or median age in the cohort. None of the participants were self-employed or had any type of DP at inclusion [14].

For each of the 213 individuals in the cohort, data were obtained on all sickness absence (including date of start and end of sick leave) and the diagnoses for each sick-leave period (n = 7,878) in each sick-leave spell (n = 4,610) from 1^st ^January 1985 to 1^st ^September 1996. In as much as the Social Insurance Offices were not allowed to store information on sick-leave diagnoses in computer records, that information was retrieved manually from each sickness certificate filed at the insurance offices. For subjects that had moved to other regions during the 11-year study period, great effort was made to find all their sickness certificates filed at the relevant local insurance offices [14]. We also collected the diagnoses reported for self-certified sick-leave spells, that is, for which a physician-issued sickness certificate was not required.

Furthermore, data on dates of granting of DP and of death were acquired from national registers.

### Measures and analyses

The following definitions were used:

*Sick-leave period*: the length of time covered by a specific sickness certificate. A sick-leave period could be prolonged with a new sickness certificate, in which the individual could also have another sick-leave diagnosis.

*Sick-leave spell*: an uninterrupted time of length of sickness absence, which could include one or several sick-leave periods.

The sick-leave diagnoses were classified according to the 18 chapters of ICD-8 (International Classification of Diseases, Revision 8) [18]. Six percent of the sickness certificates (n = 471) lacked a diagnosis but included dates, and in most cases, the name of the certifying physician and/or clinic. A plausible chapter of diagnosis was given to those periods by considering the diagnoses for the sick-leave periods before and after in the same sick-leave spell, and the clinic at which the certifying physician worked. This task was done separately but in parallel by two researchers, one a physician who was well acquainted with the different regional clinics and the other a medical student. They agreed on the classification of all but 61 of such certificates, and those 61 were assigned to the category "Symptoms, signs, and ill-defined conditions" (ICD-8 Chapter XVI). Up to 1992, all people employed by the state with sick-leave spells < 14 days received compensation directly from their employers, and thus their sickness certificates were not sent to the Social Insurance Offices. In our study, that meant that no information on diagnoses was available for 169 sick-leave periods, which were consequently also classified as "Chapter XVI."

In some analyses, the sick-leave diagnoses were categorized into three groups: musculoskeletal, mental, and all other diagnoses.

The following measures of sickness absence were used in the present study:

- Number and mean number of sick-leave days and of sick-leave periods, in total and for each diagnostic chapter.

- Frequency of sick-leave periods per person.

- Number of individuals with one or several sick-leave periods ordered according to diagnostic chapters.

- Number of individuals with only musculoskeletal diagnoses and those with a combination of musculoskeletal, mental, and all other diagnoses.

Each member of the cohort was followed until date of DP, of emigration, of death, or to end of follow up 31 December 1996.

### Sickness insurance in Sweden 1985–1996

In Sweden, all peoples aged 16–64 years are covered by the national sickness insurance, which entitles to benefits when work capacity is reduced by an illness or injury. If such incapacity is long-lasting or permanent, the claimant can be granted DP (equivalent to incapacity benefit in the United Kingdom or social security disability insurance in the United States). A full DP benefit amounts to at least 65% of previous income from work. DP is supposed to be considered after 12 months of sickness absence. However, since there was no limit to the duration of sick-leave spells in Sweden, some lasted for much longer time

It was possible to self-certify the first seven days of a sick-leave spell; after that time, a sickness certificate issued by a physician was required. Starting in 1992, compensation for the first 14 days of a sick-leave spell was paid by the employer and thus was not registered at the Social Insurance Office. Therefore, information from sick-leave spells shorter than 14 days was not included for any of the participants in the last years of the follow-up.

It has been argued that there are gender differences in sickness absence are due to that women sometimes take sick leave to care for (sick) children. This is probably not the case in Sweden, where parental insurance is generous. During the present study period, parental benefits covered absence from work to care for infants (450 days/child) and sick children (60 days/year/child). Data on this type of absence were not included in the analyses. There were no differences in levels of sickness, maternity, and parental benefits, which generally corresponded to 80% of income from work.

### Statistics

Number and mean number of sick-leave days and sick-leave periods during the follow-up (1985–1996) ordered according to the different ICD-8 diagnostic chapters, were calculated. Also, 95% confidence intervals (CI) were computed for number of days/sick-leave period in 1985–1996 recorded in the various diagnostic chapters. A bar chart was created to study the number of different sick-leave diagnoses each individuals had during the 11-year follow-up. Descriptive statistic was used to examine distribution of sick-leave periods within each diagnostic chapter for persons with ≥ 11 sick-leave diagnoses.

The study was approved by the Swedish National Data Inspection Board and the Regional Research Ethics Committee.

## Results

Over the 11-year follow-up, the young adults in this cohort had a total of 176,825 sick-leave days constituting 7,878 sick-leave periods (in 4,610 sick-leave spells) with diagnoses in 17 of the 18 ICD-8 chapters (Table 1). The only chapter not represented was Chapter XV (perinatal conditions). By far the largest proportion of sick-leave days (66%) was due to musculoskeletal diagnoses (Chapter XIII) followed by mental diagnoses (9%, Chapter V). Also, most sick-leave periods were due to musculoskeletal diagnoses (40%) followed by respiratory diseases (17%, Chapter VIII). The largest numbers of days per sick-leave period were found for the two categories neoplasm (Chapter II; 52 days/period) and mental diagnoses (47 days/period).

**Table 1:** The distribution of total number of sick days, sick-leave periods, and days/sick-leave period in 1985–1996 according to different ICD-8 chapters in a cohort of young adults initially sick-listed ≥ 28 days due to back, neck, or shoulder diagnoses

ICD-8		Sick days		Sick-leave periods		Days/sick-leave period (95% CI)	
*Chapter*	Code listing	n	%	n	%	n	95% CI

I	Infectious and parasitic diseases	3,987	2.25	789	10.02	5.05	(4.64–5.47)
II	Neoplasms	1,413	0.80	27	0.34	52.33	(29.60–75.08)
III	Endocrine, nutritional, and metabolic diseases	1,423	0.80	39	0.50	36.49	(12.69–60.28)
IV	Diseases of blood and blood-forming organs	17	0.01	1	0.01	17.00	-
							
V	Mental disorders	16,754	9.47	357	4.53	46.93	(38.69–55.17)
VI	Disease of the nervous system and sense organs	3,105	1.76	150	1.90	20.70	(7.75–33.65)
VII	Diseases of the circulatory system	995	0.56	48	0.61	20.73	(16.03–25.43)
VIII	Diseases of the respiratory system	7,810	4.42	1296	16.45	6.03	(5.56–6.49)
IX	Diseases of gastrointestinal system	3,075	1.74	171	2.17	17.98	(11.71–24.26)
X	Diseases of genitourinary system	1,545	0.87	194	2.46	7.96	(6.93–9.00)
XI	Complications of pregnancy, childbirth, and the puerperium	3,235	1.83	174	2.21	18.59	(15.73–21.46)
XII	Diseases of the skin and subcutaneous tissue	2,166	1.22	59	0.75	36.71	(8.15–65.27)
XIII	Diseases of the musculoskeletal system and connective tissue	117,139	66.25	3155	40.05	37.13	(34.39–39.86)
XIV	Congenital anomalies	39	0.02	3	0.04	13.00	(-6.72–32.72)
XV	Certain conditions originating in the perinatal period	-	-	-	-	-	-
							
XVI	Symptoms, signs, and ill-defined conditions	5,953	3.37	1022	12.97	5.82	(4.60–7.05)
XVII	Injury, poisoning, and certain other consequences of external causes	7,956	4.50	364	4.62	21.86	(18.14–25.57)
XVIII	Factors influencing health status and other contacts with health services	213	0.12	29	0.37	7.34	(3.12–11.57)
							
Total		176,825	100	7878	100	22.45	(21.16–23.73)

**Figure 1:**
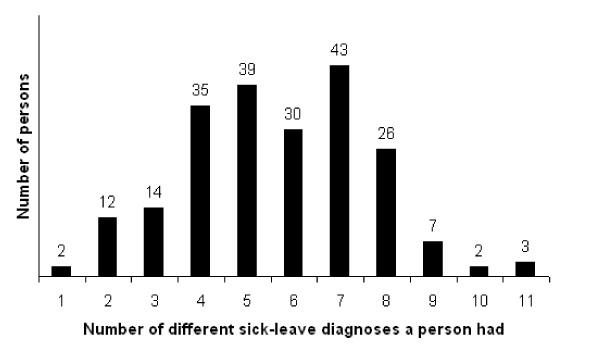
**Number of different sick-leave diagnoses**. Number of individuals who have had one to eleven different sick-leave diagnoses during the 11-year follow-up.

Over the follow-up, most of the subjects had had sick-leave periods due to four to eight different diagnoses (Figure 1), which included diagnoses such as musculoskeletal disorders (Chapter XIII), followed by respiratory diseases (Chapter VIII), symptoms, signs, and ill-defined conditions (Chapter XVI), and infectious and parasitic diseases (Chapter I) (Table 2).

**Table 2:** Distribution of number of sick-leave periods in each diagnostic category shown for persons with one to eleven different sick-leave diagnoses during the 11-year follow-up

		**Number of sick-leave periods in the ICD-8 categories (Chapters I**-**XVIII)^1^**																	
**Number of sick-leave diagnoses**	Number of subjects	I	II	III	IV	V	VI	VII	VIII	IX	X	XI	XII	XIII	XIV	XV	XVI	XVII	XVIII

**1**	n = 2													16					

**2**	n = 12	6						1	9					79			49	1	

**3**	n = 14	12				2	2	3	33			2		28			58	1	

**4**	n = 35	63				35	1	1	125	25	4	10		439			100	15	

**5**	n = 39	166				6	24	5	301	5	15	24	1	526			211	62	2

**6**	n = 30	109			1	85	19	3	154	14	8	31	15	427			146	71	

**7**	n = 43	211	12	6		99	42	2	332	59	82	48	15	789			212	86	10

**8**	n = 26	168		15		93	38	20	240	37	41	34	17	699			154	80	11

**9**	n = 7	28	15			14	21	1	49	5	20	18	5	235	3		30	11	2

**10**	n = 2	9				21	2	1	11	4	11	3	2	34			32	7	

**11**	n = 3	17		18		27	2	11	44	22	13	4	5	58			30	6	4

**Total**	n = 213	789	27	39	1	357	150	48	1296	171	194	174	59	3155	3	0	1,022	364	29

^1^See Table 1 for explanation of ICD-8 Chapters I-XVIII.

Table 3 presents distribution of the number of sick-leave periods in each ICD-8 chapter for each of the 12 subjects who had had nine, ten, or eleven different sick-leave diagnoses. Among those individuals, besides musculoskeletal disorders, respiratory diseases and symptoms, the most common sick-leave diagnoses were in the category of symptoms, signs and ill-defined conditions (Chapter XVI).

**Table 3:** Distribution of sick-leave periods in each diagnostic category shown for persons who have had nine, ten, or eleven different sick-leave diagnoses

		**Number of sick-leave periods within each diagnostic category (ICD-8 Chapters I-XVIII)^1^**																	
		
**Number of sick-leave diagnoses**	Subjects	I	II	III	IV	V	VI	VII	VIII	IX	X	XI	XII	XIII	XIV	XV	XVI	XVII	XVIII
	*A*	8				5	6		10	1				88	3		2	3	
	*B*	5	15				1		8	1	6			42			5		1
**9**	*C*	2				2	5	1	13				1	28			8	4	
	*D*	5					1		6	2	4	5	1	5			3		
	*E*	5				2			5	1	3	8		12			5		1
	*F*	2					4		5		6	4	3	4			4	2	
	*G*	1				5	4		2		1	1		56			3	2	
	
**10**	*H*	5				20	2		5	1	1		1	3			16	7	
	*I*	4				1		1	6	3	10	3	1	31			16		
	
	*J*	9				5	1		5	13	7		2	45			18	1	3
**11**	*K*	3		15		22		11	31	1	4		2	11			6	5	
	*L*	5		3			1		8	8	2	4	1	2			6		1
	
**Total**	**12**	**54**	**15**	**18**		**62**	**25**	**13**	**104**	**31**	**44**	**25**	**12**	**327**	**3**	**0**	**92**	**24**	**6**

^1 ^See Table 1 for explanation of ICD-8 Chapters I-XVIII.

During the follow-up, the results concerning sick-leave diagnoses in the three main categories (musculoskeletal, mental, and all other diagnoses) indicated that two subjects had been sickness absent solely due to musculoskeletal disorders in a total of 16 sick-leave periods, 157 had had sick-leave diagnoses including musculoskeletal and all other diagnoses, and 54 subjects had had sick-leave diagnoses including all three main categories. None of the cohort members had only the diagnostic combination of musculoskeletal and mental disorders.

## Discussion

Thus far, our study represents one of very few attempts to describe the prospective panorama of sick-leave diagnoses in a cohort of young adults who were initially sick-listed for ≥ 28 days due to neck, shoulder, or back diagnoses. As expected, we found that musculoskeletal disorders were the diagnostic category underlying most of the sick-leave periods and sick-leave days. Nevertheless, members of the study population had also been sick listed due to several other diagnoses, most of them classified in four to eight, and in some cases up to 11, of the 18 chapters of the ICD-8. Among those with diagnoses specified in more than nine chapters, their sick-leave periods were most often categorized as being the result of musculoskeletal disorders, respiratory diseases, or injury and poisoning. No more than two members of the cohort (1%) had had sick-leave periods due solely to musculoskeletal disorders, the diagnoses used for inclusion in the study. None had only the diagnostic combination of musculoskeletal and mental disorders.

### Methodological considerations

Strengths of the study are the long follow-up period, the large and detailed data set, the population based and prospective study design, and the high quality of data. In our investigation, information on sickness absence was based on register data, and was not self-reported as has been the case in many other studies in this area [1]. Start and end dates of sick-leave spells were obtained from the Social Insurance Offices, which can be regarded as a highly accurate and valid sources. The data set we used is unique, since we do not know of any other cohort study that has included such detailed information covering 11 years of sick-leave diagnoses and spells.

We conducted a population-based study that included all inhabitants of a large municipality who met the inclusion criteria. Thus, all residents with the type of sickness absence in focus were included, and there was no bias with regard to type of workplace, occupation, or clinic, which is often the case in investigations examining sickness absence both in general and in relation to specific diagnoses [1,6]. The municipality chosen is considered to be representative of many Swedish cities [6], however, the present results cannot be generalized to age groups other than that considered here. A limitation of the study was that the cohort was fairly small in size, although it was large enough to achieve our goal of analyzing the extensive number of included sick-leave periods and days.

The types of analyses we conducted require detailed information about diagnoses for each sick-leave period. Most studies of sick-leave diagnoses performed thus far have not had access to such data and have instead used the first diagnosis given for the first sick-leave period of a sick-leave spell [19] or the diagnoses for the last period in a sick-leave spell [20,21], and both those strategies have limitations. In the present investigation, data on diagnoses were retrieved manually from sickness certificates by one of the researchers in the group. For subjects that had moved to other parts of Sweden during the study period, great effort was made to find all their sickness certificates at the relevant insurance offices. This task was very time consuming, a factor that contributed to the uniqueness of our database.

The validity of the diagnoses on sickness certificates has been discussed previously [1], and the reliability of the data set we used has been tested and found to be acceptable by other investigators [22]. It is likely that more stigmatizing diagnoses, such as mental disorders, have greater validity compared to other diagnoses; in other words, most people with sick notes indicating mental diagnoses probably actually have such disorders [1].

In the present study, more than one diagnosis was listed on 21% of the sickness certificates, but only the first diagnosis was included in the analyses, because it was presumed to represent the main reason for reduced work ability. Nevertheless, in some cases of comorbidity, it can be difficult to ascertain which diagnosis has had the greatest impact on the work incapacity. It is also plausible that there is a tendency for physicians not to change the first diagnosis, even if other diagnoses are also found to be applicable when prolonging certification of a sick-leave period. This is a possible source of bias in our data, which would have led to underestimation of the variation in the diagnostic panorama.

Longitudinal data are needed to describe the patterns of sickness absence in specific diagnostic groups and to provide a better understanding of pathogenesis. Clearly, eleven years is a sufficient amount of time to study prolonged sickness absence.

We chose to investigate people sickness absent with back, neck, and shoulder disorders, because to date they represent the most common sick-leave and DP diagnoses [1]. Furthermore, we chose to study a cohort of young adults due to the pronounced economic and personal consequences of long-term sickness absence or DP early in life. Also, a population-based cohort was used in order to obtain more generalizable data. Research focused on the panorama of possible sick-leave diagnoses requires special methodological considerations. A particularly important aspect is the fact that sickness absence can be certified by different diagnoses, and in our analysis we used detailed information about diagnoses for each sick-leave period.

#### Panorama of sick-leave diagnoses

From a medical point of view, knowledge about the prognoses for future sickness absence, including the possible panorama of sick-leave diagnoses when sickness certifying a patient is highly important. There is a need for scientific evidence and more research on the pattern and type of diagnoses over time in order to elucidate the factors that influence prolonged sick leave and DP. Back and neck disorders have been the most common causes of both short- and long-term sickness absence in Sweden for many years, which has resulted in highly frequent use of health care by individuals with such disorders, and in some cases the actual ill health of such patients has been questioned. The current findings indicate that many young adults who initially had long-term sickness absence due to neck, shoulder, or back disorders have a wide range of ill health. Our description of sick-leave diagnoses over time showed that only two persons in the cohort (1%) had been sick-listed due solely to musculoskeletal disorders and the remaining 99% also had had other sick-leave diagnoses in addition to musculoskeletal disorders. The ICD-8 chapter V, comprising "diseases of the musculoskeletal system and connective tissue" is, of course, much more comprehensive than the range of diagnoses used to determine inclusion in the present study, which also might imply a broader panorama of the ill health for the cohort. Altogether, 54 individuals had a combination of musculoskeletal, mental, and other diagnoses.

It is difficult to compare our findings with those of other studies because of the scarcity of national and international research concerning the panorama of sick-leave diagnoses over time. Gjesdal et al. [23] found that in addition to socio-demographic factors, medical information predicted subsequent transition to DP among those who had been sick listed for 6–8 weeks.

Our investigation also indicated that the pattern of sick-leave diagnoses varied somewhat with the type of measure of sickness absence that was used, which agrees with several previous studies [1,24,25]. At the group level, most sick-leave days were due to musculoskeletal or mental diagnoses, whereas most sick-leave periods were the result of musculoskeletal diagnoses followed by respiratory diseases, symptoms, signs, and ill-defined conditions, and infectious and parasitic diagnoses.

For several decades, the largest diagnostic groups related to long-term sick leave have been musculoskeletal and mental disorders, and, not surprisingly, the same categories of diagnoses have also been the dominated as DP diagnoses. However, little is known about the association between musculoskeletal and mental disorders in sickness absence research. Most information on the medical aspects of sick leave arises from the main diagnosis for granting DP. In many countries in recent years, self-reported mental disorders have increased, as have episodes of sickness absence with such diagnoses [2,24], which have become the major cause of sick leave in the United Kingdom [25]. In the present cohort, mental disorders represented the second most common diagnostic category legitimating sick-leave days; in all, mental diagnoses were given for 16,754 sick-leave days in 357 sick-leave periods (mean 47 days/period). The influence of mental disorders in transition to long-term sickness absence has been highlighted in previous investigations. Gjesdal and Bratberg [26] conducted a three-year cohort study that included approximately 10% of people on long-term sick leave (>eight weeks) in Norway, and they found significantly poorer prognoses for those with mental sick-leave diagnoses and diseases affecting the nervous, respiratory, or circulatory system than for those on long-term sickness absence due to musculoskeletal disorders. Shiels et al. [25] investigated sickness certification in general practice in the United Kingdom and found that mild mental disorders accounted for almost 40% of the certified absence. Those researchers reported that relatively few patients changed from physical to mental diagnoses during a sick-leave spell. That observation is supported by our findings that, during the 11-year follow-up, almost 70% of the young adults we studied had, in addition to musculoskeletal disorders, sick-leave diagnoses other than mental illnesses, and none had only musculoskeletal and mental diagnoses to certify their sickness absence.

## Conclusion

Although the young adults we studied were initially sick listed due to back, neck, or shoulder diagnoses, under the 11-year follow-up they exhibited sickness absence certified as being due to by a wide variety of other medical diagnoses. It is possible that sickness absence with neck, shoulder, or back disorders actually comprises a wider range of ill health than is usually assumed.

## Competing interests

The authors declare that they have no competing interests.

## Authors' contributions

MV participated in the design, analysis and interpretation of data. MV wrote the manuscript. JH substantial contributed to the data treatment and interpretation of data. KA substantial contributed to the design, data collection and participated in analysis, interpretation of data and helped to draft the manuscript. All authors read and approved the final manuscript.

## Pre-publication history

The pre-publication history for this paper can be accessed here:


